# Effect of Sewer Gas

**Published:** 1872-11

**Authors:** 


Effect of Sewer Gas.—Investigation into the probable cause of the sickness which has just caused the closing of the Rhode Island conference seminary, at Greenwich, R. I., has resulted in the discovery of a leak in the sewer, in the rear of a boarding house, caused by rats, by which a portion of the sink drainage was deposited under the rear of the house.—*Med. and Surg. Reporter.*

